# Long-Term Outcomes in Hemodialysis Patients According to Combined NT-proBNP and Galectin-3 Biomarker Profiles

**DOI:** 10.3390/jcm15031129

**Published:** 2026-02-01

**Authors:** Anca Elena Stefan, Adrian Covic, Maria Alexandra Covic, Gianina Dodi, Mugurel Apetrii, Mihai Onofriescu, Simona Hogas, Stefan Iliescu, Luminita Voroneanu

**Affiliations:** 1 Faculty of Medicine, Grigore T. Popa University of Medicine and Pharmacy, University Street No. 16, 700115 Iasi, Romania; anca-elena.stefan@umfiasi.ro (A.E.S.); adrian.covic@umfiasi.ro (A.C.);; 2 Department of Nephrology, Hospital “Dr. C.I. Parhon”, Carol I Boulevard No. 50, 700503 Iasi, Romania; 3 Department of Cardiology, Institute of Cardiovascular Diseases “Prof. Dr. George. I.M. Georgescu”, Carol I Boulevard No. 50, 700503 Iasi, Romania; 4 Department of Cardiology, Hospital “Sf. Spiridon”, Independentei Boulevard No. 1, 700111 Iasi, Romania

**Keywords:** biomarker profile hemodialysis, cardiovascular risk hemodialysis, mortality hemodialysis, NT-proBNP and hemodialysis, NT-proBNP gal-3 hemodialysis

## Abstract

**Background and Hypothesis:** Mortality in hemodialysis (HD) remains high and is not fully explained by traditional risk factors. Biomarkers reflecting myocardial stress and fibrosis, together with measures of vascular stiffness, may provide additional prognostic information in this population. **Methods:** We conducted a retrospective study evaluating 173 HD patients who were clinically stable and asymptomatic at baseline over a follow-up period of over 10 years. Patients were classified into four groups based on median baseline values of NT-proBNP and galectin-3 (4234 pg/mL and 28.1 ng/mL, respectively). Primary outcomes were all-cause mortality and major adverse cardiovascular events (MACE). Pulse wave velocity (PWV) was evaluated as an additional prognostic marker. **Results:** During follow-up, 76.9% of patients died. Higher NT-proBNP levels were associated with increased all-cause mortality, irrespective of galectin-3 levels, with adjusted hazard ratios of 2.58 and 1.93 compared with the reference group (*p* < 0.05). Age and PWV were independently associated with mortality risk, corresponding to a 4% increase in risk per year of age and a 6% increase per 1 m/s increase in PWV. MACE occurred in 26.8% of patients and did not differ significantly between biomarker-defined groups. **Conclusions:** In this long-term HD cohort, elevated NT-proBNP and increased arterial stiffness were independently associated with higher all-cause mortality. These findings support the complementary prognostic value of markers of cardiac stress and vascular stiffness in chronic hemodialysis patients.

## 1. Introduction

The global prevalence of chronic kidney disease (CKD) has increased substantially over the past decades, with the number of cases increasing by 104.09% in 2019 compared to 1990 [1]. Moreover, CKD is now widely recognized as one of the leading causes of death worldwide, and it is projected that by 2040, it will become the fifth leading cause of mortality worldwide [2]. Patients with end-stage renal disease (ESRD) undergoing hemodialysis (HD) experience exceptionally high cardiovascular (CV) morbidity and mortality, which is determined by a complex interplay of traditional and non-traditional factors related to the uremic milieu, including chronic volume overload, inflammation, fibrosis, oxidative stress, and vascular calcification [3]. These mechanisms contribute to myocardial remodeling, arterial stiffening, and progressive cardiovascular dysfunction, which are imperfectly captured by conventional risk scores in this population.

Galectin-3 has been studied as a potential biomarker for predicting CV risk and mortality in HD patients. Galectin-3 is a β-galactoside-binding protein that is expressed in various tissues. It is located intracellularly or secreted into the extracellular space, and it plays a role in cell proliferation, differentiation, apoptosis, fibrosis and inflammation [4]. Galectin-3 has been widely studied in CV disease, CKD, but also in rheumatological conditions, such as rheumatoid arthritis, gastrointestinal diseases and idiopathic pulmonary fibrosis [5]. However, evidence regarding its prognostic value in HD populations remains inconsistent. While meta-analyses suggest an association between galectin-3 and all-cause mortality in CKD, its predictive relevance appears attenuated or heterogeneous in patients receiving hemodialysis, particularly over longer follow-up periods [6].

NT-proBNP is a well-established biomarker of myocardial stretch and volume-related cardiac stress and has demonstrated robust prognostic value. In a previous study, our group combined NT-proBNP and galectin-3 to assess complementary biological domains—myocardial stress and fibrotic/inflammatory activity—and demonstrated that this biomarker-based approach improved the prediction of cardiovascular outcomes over a median follow-up of 3 years in clinically stable, asymptomatic HD patients [7]. An additional consideration is the time-dependent behavior of cardiovascular biomarkers. In HD population, long-term outcomes may predominantly reflect cumulative structural myocardial and vascular alterations, potentially modifying the prognostic performance of biomarkers related to distinct biological pathways.

Therefore, the present study aimed to reassess this well-characterized HD cohort over more than 10 years of follow-up to evaluate the long-term prognostic value of combined NT-proBNP and galectin-3 biomarker profiles in relation to arterial stiffness. The primary outcomes were all-cause mortality and major adverse cardiovascular events (MACE), with an exploratory analysis of mortality stratified by PWV.

## 2. Materials and Methods

This is a long-term (13 years) retrospective observational study designed to assess all-cause mortality and MACE in a cohort of patients with ESRD on intermittent hemodialysis. The cohort included 173 clinically stable, asymptomatic patients receiving standard thrice-weekly HD treatment. Patients were included if they had been on HD for at least 3 months and were recruited from two HD units in Iasi, Romania. Exclusion criteria were known ischemic heart disease (diagnosed by stress test or angiography), prevalent heart failure (defined as a documented clinical diagnosis of heart failure), active malignancy and acute infections. Arterial stiffness—PWV, was measured through applanation tonometry of the carotid and femoral arteries using SphygmoCorTM; PWV Inc., Westmead, Sydney, Australia before the dialysis session in all participants in 2012. All participants in the original study provided written informed consent, and the study was approved by the local ethics committee of “Dr. C.I. Parhon” Hospital. The current study retrospectively evaluated the initial cohort and was approved by the local Ethics Committee (approval no. 9019, 19 September 2025).

### 2.1. Follow-Up and Data Collection

We conducted a long-term observational study (February 2012–December 2025) to evaluate survival and cardiovascular outcomes in the initial cohort. Detailed longitudinal data on dialysis adequacy (Kt/V) and residual renal function were not consistently available for the entire cohort over the long duration of follow-up and therefore could not be reliably included in the analyses. Vital status (alive or deceased) was verified through dialysis center records or by ascertaining the national electronic health database. For deceased patients, the presumed cause of death was determined from available clinical documentation, including hospital discharge summaries and dialysis unit reports. When cause of death was not identified or when the cause of death was sudden death (as reported by dialysis center records), patients were excluded from the cause-specific CV mortality analysis but retained in the all-cause mortality dataset. CV events (stroke, acute myocardial infarction, coronary revascularization procedures) were identified through electronic records from “Dr. C.I. Parhon” Hospital and corresponding dialysis centers. Regarding year of death, 3 records were missing.

The classification of patients into four subgroups according to baseline Gal3 and NT-proBNP levels—Group 1: low Gal3, low NT-proBNP (Gal-3 < 28.1 ng/mL and NT-proBNP < 4234 pg/mL); Group 2: high Gal3, low NT-proBNP (Gal-3 ≥ 28.1 ng/mL and NT-proBNP < 4234 pg/mL); Group 3: low-Gal3, high NT-proBNP (Gal-3 < 28.1 ng/mL and NT-proBNP ≥ 4234 pg/mL); Group 4: high Gal3, high NT-proBNP (Gal-3 ≥ 28.1 ng/mL and NT-proBNP ≥ 4234 pg/mL) was retained from the original study. The cut-off values for Gal3 and NT-pro-BNP (28.1 ng/mL and 4234 pg/mL, respectively) were based on their respective median concentrations in the baseline cohort.

### 2.2. Outcomes

The primary outcome was all-cause mortality during follow-up. The secondary outcomes included: three-point MACE, defined as non-fatal stroke, non-fatal acute myocardial infarction and CV death. This outcome was chosen to ensure the accuracy and reliability of the data, as detailed, verified information regarding heart failure hospitalization, arrhythmias, or unstable angina was not available for the entire cohort. We aimed to minimize misclassification bias and improve the robustness of the cardiovascular outcome assessment.

An exploratory analysis was conducted to assess the association between baseline PWV and long-term all-cause mortality, with the cut-off pf PWV being the median value of the included cohort (9.85 m/s). The use of a median-based PWV cut-off (≥9.85 m/s) was limited to exploratory interaction analyses aimed at evaluating whether the prognostic impact of arterial stiffness differed across biomarker-defined subgroups. This approach was not intended to define a clinically actionable threshold, but rather to facilitate interpretation of effect modification in a long-term observational setting. Additionally, PWV was primarily modeled as a continuous variable in the all-cause mortality analysis.

### 2.3. Statistical Analysis

All statistical analyses were performed using JASP (version 0.95.4). Continuous variables were expressed as mean ± standard deviation or median and interquartile range (IQR), as appropriate. Categorical variables were summarized as absolute numbers and percentages. The normality of continuous variables was assessed using the Shapiro–Wilk test and visual inspection of histograms. Baseline differences across the four groups were assessed using one-way ANOVA for normally distributed continuous variables and Kruskal–Walli’s test for non-normally distributed continuous variables. Categorical variables were compared using χ^2^ or Fisher’s exact test, as appropriate. A two-tailed *p*-value < 0.05 was considered statistically significant.

All-cause mortality was analyzed using the Kaplan–Meier method, with comparisons between the four defined groups using the log-rank test. Cox proportional hazard regression was used to identify predictors for all-cause mortality. Covariates included age, sex, diabetes, hypertension, peripheral artery disease, dialysis vintage, PWV and the four defined groups. Results were reported as hazard ratios (HR) with 95% confidence intervals (CI). The proportional hazards assumption was verified using scaled Schoenfeld residuals and model-calibration was assessed through visual inspection of log-minus-log plots. MACE was analyzed as a binary outcome using logistic regression. The primary model included the same covariates in the Cox model. Model was assessed using the Hosmer–Lemeshow test and Nagelkerke’s R^2^. A sensitivity analysis was performed using a parsimonious model (including only age, sex, diabetes and groups). The stability of estimates was evaluated by comparing odds ratios (OR) between models. PWV was treated as a continuous variable (per 1 m/s increase) and as a categorical variable dichotomized at 9.85 m/s (the median value of the cohort). Sensitivity analyses were conducted to ensure the model’s robustness.

## 3. Results

### 3.1. Baseline Characteristics

The cohort included 173 patients. Baseline demographic, clinical, biological and vascular characteristics of the entire population are presented in Table 1. The median levels of Gal-3 and NT-proBNP were 28.1 (ng/mL, IQR: 18.7–40.4 ng/mL) and 4234 pg/mL (IQR: 1826.5–11.581 pg/mL), respectively. The patients were divided into four groups based on median Gal-3 and NT-proBNP levels:

- Group 1: low Gal-3/low NT-proBNP (Gal-3 < 28.1 ng/mL and NT-proBNP < 4234 pg/mL) (*n* = 44);
- Group 2: high Gal-3/low NT-proBNP (Gal-3 ≥ 28.1 ng/mL and NT-proBNP < 4234 pg/mL) (*n* = 43);
- Group 3: low Gal-3/high NT-proBNP (Gal-3 < 28.1 ng/mL and NT-proBNP ≥ 4234 pg/mL) (*n* = 43);
- Group 4: high Gal-3/high NT-proBNP (Gal-3 ≥ 28.1 ng/mL and NT-proBNP ≥ 4234 pg/mL) (*n* = 43).

**Table 1:** Baseline characteristics.

	All (n = 173)	Group 1 (N = 44)	Group 2 (N = 43)	Group 3 (N = 43)	Group 4 (N = 43)	*p* *
**Age, years**	58.6 ± 14.8	59.0 ± 13.9	55.8 ± 16.7	57.7 ± 14.0	61.8 ± 14.2	0.29
**Male, n (%)**	84 (48.6)	22 (50.0)	26 (60.5)	15 (34.9)	21 (48.8)	0.13
**Dialysis vintage, months**	42.9 (17.6–89.3)	28.1 (13.9–70.4)	36.2 (16.6–114.6)	55.3 (20.9–102.2)	48.1 (20.5–80.9)	0.17
**Anuric, n (%)**	91 (52.6)	20 (45.5)	24 (55.8)	23 (53.5)	24 (55.8)	0.74
**Hypertension, n (%)**	140 (80.9)	35 (79.5)	34 (79.1)	37 (86.0)	34 (79.1)	0.83
**Diabetes, %**	29 (16.8)	10 (22.7)	5 (11.6)	7 (16.3)	7 (16.3)	0.60
**PAD, n (%)**	23 (13.3)	7 (15.9)	5 (11.6)	6 (14.0)	5 (11.6)	0.96
**PWV (m/s)**	9.85 (7.6–11.4)	9.49 (8.4–11.5)	9.78 (8–10.7)	9.95 (8.3–11.6)	10.21 (8.6–11.9)	0.793

PAD: peripheral arterial disease, PWV: pulse wave velocity, * *p* values were calculated using one-way ANOVA for normally distributed continuous variables, Kruskal–Wallis test for non-normally distributed variables, and chi-square test for categorical variables.

### 3.2. Primary Outcome—All-Cause Mortality

Over a median follow-up of 13 years, 76.9% of patients died. Among patients with known causes of death, 21.1% died from CV causes, 21.1% from sepsis, and 22.6% from other causes (pulmonary embolism, terminal illness, or intracerebral hemorrhage). Sudden death occurred in 31.6% of all recorded deaths. The cause of death remained undetermined in 3.6% of cases.

**Figure 1:**
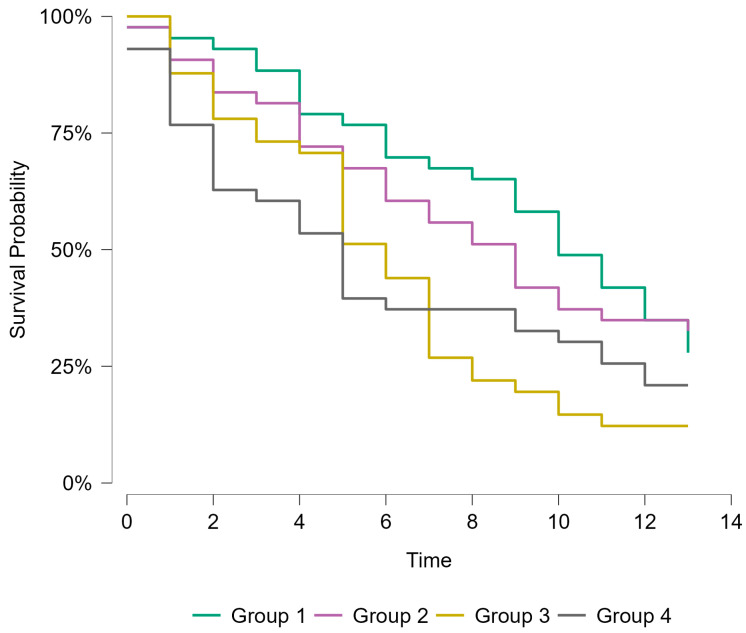
Kaplan–Meier survival analysis—All-cause mortality.

Kaplan–Meier survival analysis demonstrated significant differences in all-cause mortality across the four biomarker-defined groups (log-rank χ^2^ = 10.6, *p* = 0.014). Survival was longest in Group 1 and progressively decreased across Groups 2 to 4, indicating a stepwise worsening of prognosis relative to Group 1. Patients with elevated levels of NT-proBNP ≥ 4234 pg/mL (groups three and four) demonstrated the poorest survival rate compared to those with low levels of NT-proBNP (groups one and two). Group 4 had the shortest median survival time (5 years, 95% CI, 2–10), followed by group 3 (6 years, 95% CI, 5–7). Survival curves diverged early during follow-up, with the greatest separation observed after 5 years (Figure 1).

In the unadjusted Cox proportional hazards model, Group 1 was used as the reference category. Compared with Group 1, patients in Group 3 and Group 4 had a significantly higher risk of all-cause mortality (HR = 1.95, 95% CI 1.20–3.16, *p* = 0.007; and HR = 1.70, 95% CI 1.04–2.76, *p* = 0.034, respectively). Group 2 did not differ significantly from the reference group (HR = 1.07, 95% CI 0.65–1.78, *p* = 0.79).

These results suggest that a profile characterized by NT-proBNP ≥ 4234 pg/mL, alone or in combination with high Gal-3 levels, is associated with increased long-term mortality in HD patients (Table 2).

Next, a multivariable Cox proportional hazards model was performed to identify independent predictors of all-cause mortality. The model was adjusted for age, sex, presence of diabetes mellitus, arterial hypertension, peripheral artery disease, pulse wave velocity and dialysis vintage (months).

After adjustment, both Group 3 and Group 4 remained independently associated with mortality (HR 2.58 [1.54–4.32], *p* < 0.001; HR 1.93 [1.17–3.20], *p* = 0.011). Group 2 did not differ significantly from the reference.

Among the covariates, both age and PWV were independently associated with higher mortality risk. Each one-year increase in age was associated with a 4% higher risk of death (HR 1.04 [95% CI 1.03–1.06], *p* < 0.001). On the other hand, each 1 m/s increase in PWV corresponded to a 6% increase in mortality risk (HR 1.06 [95% CI 1.01–1.11], *p* = 0.019). Arterial hypertension showed a borderline protective effect (HR 0.63 [95% CI 0.40–1.00], *p* = 0.050). Other covariates (diabetes mellitus, peripheral artery disease, sex and dialysis vintage) were not associated with an increase in mortality after adjustment (Table 3).

#### Sensitivity Analysis

Proportional hazards assumptions were assessed using scaled Schoenfeld residuals. The global test indicated a mild departure from proportionality (χ^2^ = 19.37, df = 10, *p* = 0.036), primarily driven by age (*p* = 0.006) and group (*p* = 0.029). All other covariates met the proportional hazards assumption (all *p* > 0.050).

Importantly, visual inspection of scaled Schoenfeld residual plots did not reveal systematic time-dependent effects. Additional diagnostic analyses based on deviance and influence residuals showed no influential outliers or leverage points and demonstrated a symmetrical distribution of residuals.

Given the long duration of follow-up and the complexity of the multivariable model, these findings were considered acceptable, and the proportional hazards assumption was regarded as reasonably met. Overall, results indicate good model stability and robustness.

### 3.3. Secondary Outcome—MACE

For MACE analysis, data was available for 127 patients after excluding patients with sudden or death of undetermined cause. During follow-up, 34 MACE (26.8%) were recorded: 79.4% cardiovascular deaths, 14.7% non-fatal myocardial infarctions, and 5.9% non-fatal strokes. In the unadjusted logistic regression model excluding patients with sudden death, no statistically significant differences in MACE risk were observed across the predefined groups (*p* = 0.709). Compared to group 1, the odds ratios for MACE were 0.70 (95% CI 0.23–2.10, *p* = 0.53) for Group 2, 0.53 (95% CI 0.16–1.72, *p* = 0.28) for Group 3, and 0.88 (95% CI 0.30–2.58, *p* = 0.8). Model fit indices indicated no improvement in predictive power compared to the null model (Δχ^2^ = 1.37, *p* = 0.71; Nagelkerke R^2^ = 0.016).

To test the robustness of our findings, we performed a multivariate logistic regression including patients who experienced sudden death and in patients for whom cause of death was unspecified (n = 173). In this second analysis, results remained directionally consistent with the primary model, although none of the predictors reached statistical significance (global model *p* = 0.42, Nagelkerke R^2^ = 0.09). Peripheral artery disease and diabetes remained positively associated with MACE risk, whereas PWV and biomarker-defined groups showed no significant association.

After adjusting for demographic, vascular, and dialysis-related factors, peripheral artery disease emerged as the leading independent predictor of MACE, with patients showing a 5.5-fold higher risk compared to those without PAD. Diabetes was more frequent in patients with adverse outcomes, but this relationship did not reach statistical significance. No independent association was found for the biomarker-defined groups or PWV (Table 4).

### 3.4. Exploratory Outcomes

A semi-parametric Cox proportional hazards model including group, PWV, and their interaction was fitted to assess the differential impact of arterial stiffness across biomarker-defined subgroups. The analysis included 170 patients and 130 events. Model comparison showed a slight improvement in fit (AIC = 1179.18 vs. 1179.80). The global proportional hazards test revealed mild deviation (χ^2^ = 19.37, df = 10, *p* = 0.036), but the model was considered acceptable.

In the multivariate Cox model including the defined groups and PWV and their interaction, results indicated that a PWV **≥** 9.85 m/s was independently associated with all-cause mortality (HR 3.1, 95% CI 1.51–6.34, *p* = 0.002). A significant interaction between Group 3 and PWV (*p* = 0.040) was observed, suggesting that the effect of arterial stiffness on mortality differed across biomarker-defined groups. In Group 4 (both biomarkers elevated), mortality was already high irrespective of arterial stiffness (Table 5).

**Figure 2:**
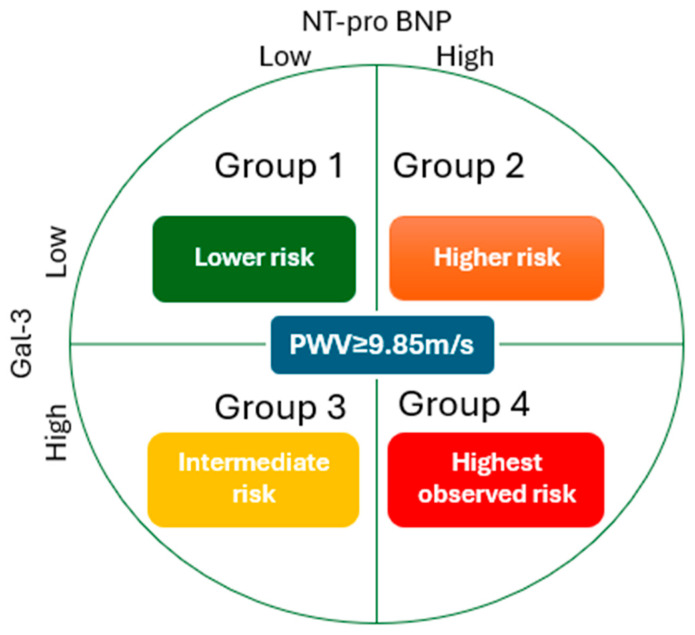
Mortality risk among biomarker-defined groups and PWV.

In summary, long-term mortality in this HD cohort was primarily driven by markers of myocardial stress and arterial stiffness, namely NT-proBNP and PWV. In contrast, gal-3 did not retain independent prognostic value over extended follow-up. Although biomarker-defined differences in survival were observed, no significant associations were identified between biomarker profiles and three-point MACE. Figure 2 illustrates the four biomarker-defined groups based on baseline NT-proBNP and galectin-3 levels, with colors indicating relative long-term mortality risk within the study population rather than absolute clinical thresholds.

## 4. Discussion

In this long-term observational study of clinically stable hemodialysis patients, we evaluated the prognostic relevance of NT-proBNP and galectin-3 in relation to arterial stiffness over more than a decade of follow-up. The principal finding was that elevated NT-proBNP was consistently associated with increased all-cause mortality, whereas the long-term prognostic contribution of galectin-3 appeared limited. Minor departures from the proportional hazards assumption were observed, mainly for age and group; however, visual diagnostics did not indicate meaningful time-dependent effects, and overall model stability was considered acceptable. In this cohort, arterial stiffness, assessed by pulse wave velocity (PWV), emerged as an independent determinant of mortality risk, with a more pronounced effect observed in patients with high NT-proBNP and low galectin-3 levels.

In our previous 3-year follow-up study, NT-proBNP and galectin-3 were both linked with CV outcomes in stable, asymptomatic HD patients [7]. The present findings expand on these observations by demonstrating that NT-proBNP retains prognostic relevance over extended follow-up in a stable cohort with minimal loss to observation. This supports a concept framework of chronic myocardial stress as a durable risk signal in long-term hemodialysis patients, beyond short-term fluctuations in volume status. In contrast, galectin-3, a biomarker associated with inflammatory and fibrotic pathways, did not show sustained, discriminative long-term prognostic value when evaluated alongside NT-proBNP. Although galectin-3 has been linked to cardiovascular outcomes in shorter-term studies, its predictive value may attenuate over time, potentially reflecting dynamic biological regulation, therapeutic modulation, or competing risk factors in chronic dialysis populations [8].

Previous meta-analyses in CKD and HD cohorts have reported heterogenous associations between galectin-3 and mortality. Zhang et al. [6] found that elevated galectin-3 was associated with all-cause mortality in CKD, while its prognostic value in HD remained uncertain. More recent data, including the meta-analysis by Bellos et al. [9], have confirmed that galectin-3 levels above 30 ng/mL are associated with a higher mortality risk. However, this connection was less established in HD than in pre-dialysis CKD. Our findings are consistent with these observations and suggest that in a long-term hemodialysis setting, galectin-3 alone may provide limited incremental prognostic information beyond NT-proBNP.

NT-proBNP and galectin-3 reveal different but complementary biological pathways. NT-proBNP mainly reflects myocardial stretch, cavity pressure, and volume overload, although galectin-3 is involved in macrophage activation, myocardial fibrosis, and systemic inflammation [10]. In dialysis patients, chronic exposure to fluid overload, uremic toxins, and repetitive myocardial ischemia–reperfusion episodes cause both cardiac remodeling and vascular stiffening [11]. Galectin-3 may function primarily as an intermediate marker—its elevation may reflect tissue remodeling but inevitably does not predict long-term outcomes once structural cardiac damage is recognized [12]. In end-stage renal disease, marked elevation of galectin-3 may reduce long-term discriminatory capacity, whereas long-term mortality in hemodialysis appears to be driven by persistent myocardial stress and vascular stiffness, explaining the attenuation of galectin-3’s independent prognostic value [9]. This hypothesis aligns with mechanistic studies showing that galectin-3 inhibition in animal models reduces fibrosis but does not reverse established structural abnormalities [13].

Arterial stiffness, quantified by PWV, provided complementary prognostic information independent of biomarker profiles. Increased PWV likely reflects cumulative vascular damage related to uremia, vascular calcification, and aging, integrating long-term structural arterial changes that are not fully reflected by circulating biomarkers [14]. The independent association between PWV and mortality underscores the relevance of vascular dysfunction as a parallel contributor to cardiovascular risk in hemodialysis.

PWV has consistently been associated with mortality in hemodialysis; however, its integration with biomarker-based risk assessment has not been frequently explored [15]. Our results suggest that combining PWV with NT-proBNP may help identify patients at particularly high long-term risk. This approach could potentially refine risk stratification beyond conventional clinical variables, especially in asymptomatic individuals without overt cardiovascular disease. Importantly, no significant differences in major adverse cardiovascular events were observed across biomarker-defined groups. This may reflect the competing risk of non-cardiovascular mortality in long-term hemodialysis cohorts, the heterogeneity of cardiovascular events, or limited statistical power for secondary outcomes.

Limitations of our study include: the cohort size was modest, and biomarker measurements were obtained at a single baseline time point. Temporal changes in NT-proBNP, galectin-3, and PWV were unavailable, limiting the assessment of dynamic risk trajectories. The high proportion of sudden deaths in our cohort may have led to an underestimation of cardiovascular-specific mortality. Although the study had an exceptionally long follow-up and minimal loss to observation, residual confounding by unmeasured factors cannot be excluded. Finally, these results are derived from a single geographic region and may not be generalizable to other populations or dialysis modalities. In addition, some effect estimates were associated with relatively wide confidence intervals, reflecting limited precision due to the modest sample size and number of events; however, extensive model diagnostics and sensitivity analyses supported the overall stability and robustness of the findings.

## 5. Conclusions

In conclusion, our findings suggest that markers of myocardial stress and vascular damage are more consistently associated with long-term mortality in hemodialysis than fibrotic or inflammatory biomarkers. This emphasizes the need to differentiate transient from sustained risk signals and to confirm these findings in larger studies.

## Figures and Tables

**Table 2:** HR derived from Cox proportional hazards regression.

Variable	Hazard Ratio	95%CI	*p*-Value
**Group 2 vs. 1**	1.071	0.645–1.777	0.791
**Group 3 vs. 1**	1.946	1.198–3.163	0.007
**Group 4 vs. 1**	1.696	1.041–2.761	0.034

**Table 3:** Multivariable Cox regression for all-cause mortality.

Variable	HR	95% CI	*p*-Value
**Group 2 vs. 1**	1.49	0.88–2.51	0.137
**Group 3 vs. 1**	2.58	1.54–4.32	0.001
**Group 4 vs. 1**	1.93	1.17–3.20	0.009
**Diabetes mellitus**	1.26	0.78–2.04	0.340
**Arterial hypertension**	0.63	0.40–1.00	0.050
**PAD**	1.08	0.64–1.80	0.782
**Sex**	0.97	0.67–1.39	0.856
**Age (per year)**	1.04	1.03–1.06	<0.001
**PWV (per 1 m/s)**	1.06	1.01–1.11	0.020
**Dialysis vintage (months)**	1.00	0.99–1.00	0.177

PAD—peripheral artery disease, PWV—pulse wave velocity.

**Table 4:** Multivariate logistic regression for MACE.

Variable	Odds Ratio (OR)	95% CI	*p*-Value
**Group 2**	0.65	0.20–2.10	0.484
**Group 3**	0.37	0.09–1.48	0.166
**Group 4**	0.78	0.27–2.23	0.661
**PAD**	5.57	1.28–24.2	0.023
**Diabetes**	4.10	0.95–17.8	0.064
**Arterial hypertension**	0.44	0.15–1.32	0.163
**Sex**	1.85	0.70–4.88	0.195
**Age**	1.09	0.93–1.28	0.273
**Dialysis vintage**	0.99	0.97–1.02	0.243
**PWV (continuous)**	1.09	0.95–1.25	0.227

PAD—peripheral artery disease, PWV—pulse wave velocity.

**Table 5:** Cox regression with PWV-Group interaction.

Groups—PWV	HR	CI	*p*-Value
**PWV ≥ 9.85 m/s**	3.10	1.51–6.34	0.002
**Group 2 × PWV**	0.74	0.26–2.07	0.560
**Group 3 × PWV**	0.36	0.14–0.95	0.040
**Group 4 × PWV**	0.55	0.20–1.46	0.230

PWV—pulse wave velocity.

## Data Availability

The original contributions presented in this study are included in the article. Further inquiries can be directed to the corresponding author.

## Abbreviations

The following abbreviations are used in this manuscript:

CKD	Chronic kidney disease
CV	Cardiovascular
Gal-3	Galectin-3
NT-proBNP	N-terminal pro-B-type natriuretic peptide
ESRD	End-stage renal disease
PWV	Pulse wave velocity
HD	hemodialysis
